# Producing Gestures Facilitates Route Learning

**DOI:** 10.1371/journal.pone.0112543

**Published:** 2014-11-26

**Authors:** Wing Chee So, Terence Han-Wei Ching, Phoebe Elizabeth Lim, Xiaoqin Cheng, Kit Yee Ip

**Affiliations:** 1 Department of Educational Psychology, The Chinese University of Hong Kong, Hong Kong, Hong Kong, S.A.R.; 2 Department of Psychology, National University of Singapore, Singapore, Republic of Singapore; University of Regensburg, Germany

## Abstract

The present study investigates whether producing gestures would facilitate route learning in a navigation task and whether its facilitation effect is comparable to that of hand movements that leave physical visible traces. In two experiments, we focused on gestures produced without accompanying speech, i.e., co-thought gestures (e.g., an index finger traces the spatial sequence of a route in the air). Adult participants were asked to study routes shown in four diagrams, one at a time. Participants reproduced the routes (verbally in Experiment 1 and non-verbally in Experiment 2) without rehearsal or after rehearsal by mentally simulating the route, by drawing it, or by gesturing (either in the air or on paper). Participants who moved their hands (either in the form of gestures or drawing) recalled better than those who mentally simulated the routes and those who did not rehearse, suggesting that hand movements produced during rehearsal facilitate route learning. Interestingly, participants who gestured the routes in the air or on paper recalled better than those who drew them on paper in both experiments, suggesting that the facilitation effect of co-thought gesture holds for both verbal and nonverbal recall modalities. It is possibly because, co-thought gesture, as a kind of representational action, consolidates spatial sequence better than drawing and thus exerting more powerful influence on spatial representation.

## Introduction

We are constantly using our ability to form and manipulate representations of space. For example, we use spatial abilities to navigate in an unfamiliar environment or find a way back home. Spatial abilities are also crucial for us to acquire abstract metaphors and analogies (e.g., [1]). Some researchers even argue that spatio-motor processing underlies all cognition, including abstract thought [2], [3]. Moreover, spatial skill is strongly associated with achievement in science, technology, engineering, and mathematics (STEM) [4]. Therefore, developing techniques to improve one's spatial abilities has received increasing attention from cognitive and educational psychologists all over the world.

A wealth of research has been done to develop learning techniques which help individuals to form spatial representations and improve their spatial skills. Those techniques involve spatial language (e.g., [5]); sketching (e.g., [6]); and maps, charts and diagrams (e.g., [7]). Besides these techniques, hand movements like *gestures* might be effective in processing and learning spatial information. As a natural accompaniment to speaking or thinking about moving through space, manual gestures are inherently spatial [8], [9]. Learners can thus exploit the spatial character of their gestures to encode the movement through space that map routes represent.

In fact, abundant research has shown that gestures convey spatial information. For example, speakers produce co-speech gestures (gestures that are co-occurring with speech) as they describe spatial arrangement of objects [10], [11] and identify spatial relation between two characters in their narratives [12], [13]. Speakers also increase gesture production when encountering difficulty in describing complex spatial patterns [14], [15].

In spite of the fact that gestures represent spatial information, there is a surprising paucity of research investigating whether producing gestures facilitates *encoding* of spatial information. There are a few studies, however, that examine the role of gestures in spatial visualization, one of the sub-skills of spatial abilities. Spatial visualization is the ability to mentally manipulate, rotate, twist, or invert objects without reference to one's self [16]. In one of the studies, Ehrlich, Levine, and Goldin-Meadow [17] found that boys solved more problems than did girls in the mental rotation task, and interestingly, boys also produced co-speech gestures more often than did girls when giving solutions. Such finding suggested a positive association between gesture production and spatial visualization.

While Ehrlich et al. [17] studied co-speech gestures, other researchers focused on co-thought gestures (gestures that are produced when thinking silently in a non-communicative context) (e.g., [18], [19]). Chu and Kita [18] found that participants spontaneously produced co-thought gestures when they were solving mental rotation tasks. More importantly, participants who were encouraged to produce co-thought gestures while solving mental rotation tasks performed significantly better than did those who were not encouraged to gesture and those who were not allowed to gesture [20].

The present study focuses on co-thought gestures and examines whether gesturing while memorizing spatial information improves recall. Recent research has shown that gesturing does enhance acquisition of new information but those studies focused on mathematical knowledge. For example, children who were told to gesture when explaining their solutions to a math problem benefited more from the subsequent math lesson than children who were told not to gesture [21]. In addition, children who were instructed to reproduce a teacher's gestures while acquiring new mathematical concepts learned better than those who were instructed to reproduce only the teacher's verbal instructions [22].

However, no experimental work to date has examined whether gestures strengthen learning of spatial information. If co-thought gestures merely convey spatial information, then participants who are told to gesture when rehearsing silently should have comparable recall to those who are told not to gesture. In contrast, if co-thought gestures are involved in encoding spatial information, then participants who are told to gesture during rehearsal should recall better than those who are told not to gesture.

Thus, the first objective of the present study was to address this issue. Adult participants in two experiments were told to study various routes and to rehearse the routes by producing co-thought gestures (e.g., index finger moves up and then to the right). We then compared their recall performance to another two groups of learners who were instructed, respectively, to rehearse the routes mentally while having their hand movements prevented and to read letters that prevented rehearsal.

Previous findings on gestural facilitation of learning in mathematics [21], [22] and spatial domains [20] also motivate us to examine whether empty-handed gestures *or* hand movements that manipulate objects are responsible for the positive learning outcome. Previous studies, however, were not able to tell the difference between these two kinds of hand movements. Thus, the second goal of the present study was to address this issue by comparing the learning outcomes of producing empty-handed gestures that do not leave visible trails (e.g., gesture a route in the air) and hand movements that leave visible trails (e.g., drawing a route on paper with a pen). Both producing co-thought gestures and drawing spatial sequences are actions as they involve hand movements. They also convey substantive information about learners' spatial representation of the routes. As opposed to drawing a route on paper, however, producing co-thought gestures during rehearsal does not leave visible physical traces of the route. One possibility is that hand movements regardless of whether they leave visible trails contribute to learning. Another possibility is gesturing in the air results in more powerful influence on spatial representation than drawing. Previous research has shown support for the second possibility. In a study by Goldin-Meadow and Beilock [23], adult participants performed the Tower of Hanoi Task in which the smallest disk was the lightest. After solving the task, one group of the participants explained how they solved it in speech and co-speech gestures (gesture group) while another group simply moved the disks (action group). Both groups used one hand to represent/lift the lightest disk. Then they solved the second Tower of Hanoi Task. Half of the participants in each group were presented with the same tower in which the smallest disk was the lightest (no-switch condition) whereas another half were given a different tower in which the smallest disk was the heaviest (switch condition). For participants in the gesture group, they improved in the second trial of the no-switch condition (took less time and made fewer moves) but declined in the switch condition. In contrast, participants in the action group had comparable performance in the two trials in both the switch and no-switch conditions. This pattern is consistent with a representation, effected via co-speech gestures, of the smallest disk as the lightest, which is helpful in no-switch trials but misleading in switch trials. Thus, producing gestures affects what we learn about a task beyond what we acquire simply by performing it.

Novack and colleagues [24] proposed that learners who act on objects while learning a concept may become preoccupied with irrelevant details (e.g., irrelevant aspects of a procedure rather than on the concept underlying it). On the contrary, learners who learn the same subject matter with gestures may focus attention on the relevant aspects of the concept. In their study, they taught children learners a strategy to solve mathematical-equivalence problems. Children were assigned to three different conditions: physical action condition in which children learnt the strategy by moving the number tiles on the board; concrete gesture condition in which children learnt by gesturing toward the number tiles without physically touching them; and abstract gesture condition in which children produced gestures under the number tiles. The results showed that children in the abstract gesture condition scored higher than those in the concrete gesture and physical action conditions in the post-test that consisted of questions comparable to those in the pre-test and in the generalization test that assessed a deeper understanding of mathematical equivalence. Hence, learning with gestures promotes conceptual understanding better than learning with concrete objects.

In the present study, we specifically asked whether empty-handed gestures have more powerful influence on spatial representation than hand movements that leave visible physical traces. If hand movements in general (i.e., with and without leaving visible physical traces) facilitate learning of spatial information, then participants who are instructed to produce co-thought gestures should have comparable recall to those who are told to draw on paper. In contrast, if empty-handed gestures are more powerful than hand movements leaving visible physical traces in learning spatial information, then participants who are told to produce co-thought gestures should have better recall than those who draw on paper. In order to test this hypothesis, adult participants in two experiments were asked to study routes and draw them on paper during rehearsal. Then we compared their recall performance to that of participants who were told to gesture.

## Experiment 1

In the first experiment, participants were presented with four routes shown in four diagrams, one at a time. Each route contained thirteen steps. Participants first learned the steps by tracing the complete routes. Then they were randomly assigned to one of the following four conditions: 1) rehearsing the routes using their preferred hand (co-thought gesture); 2) rehearsing the routes by drawing them on paper (drawing); 3) rehearsing the routes by mentally simulating them with both hands holding a ball (hand movement prevented); and 4) reading letters aloud to prevent rehearsal (no rehearsal). Then all participants verbally recalled the routes to an experimenter. We predicted that hand movements involved in co-thought gestures and drawing would facilitate encoding of spatial information, and hence enhance recall. Thus, participants in the co-thought gesture and drawing conditions should recall more steps than those in other two conditions. However, according to previous findings, we also predicted that participants who produced co-thought gestures would recall more steps than those who drew the routes on paper.

### Method

#### Participants

One hundred and twelve English-speaking undergraduates (63 men, age range: 18–23 years) participated in this experiment in order to fulfill course requirements. All of them had correct or correct-to-normal vision. Participants were asked whether they preferred using right or left hands for their daily life activities [25]. All participants were right-handed.

#### Stimuli

Four diagrams were created by the software “Edraw Max” [26]. In each diagram, there were seven vertical lines and ten strokes that were horizontal, diagonal, or curved connecting or not connecting with the vertical lines. The strokes that were connected to vertical lines formed a route navigating from the starting point to the destination. All routes were thus two dimensional static representations of an ordered sequence of actions.

**Figure 1:**
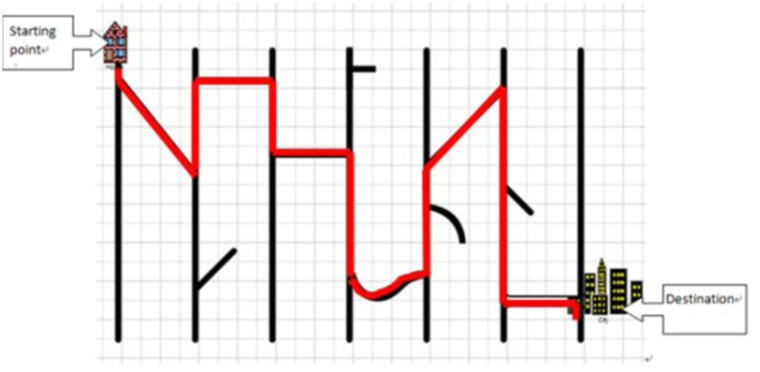
A route navigating from the starting point to the destination. The red line shows the sequence of steps.

See Figure 1 for the route (highlighted in red). In this route, one should move down, then move diagonally downwards, move up, move to the right, move down, move to the right, move down, cross the curved road, move up, move diagonally upwards, move down, cross the bridge, and finally move to the destination. Each route had thirteen steps. A step is a part of the route between vertices.

#### Procedures

Participants were randomly assigned to one of the four conditions (co-thought gesture, drawing, hand movement prevented, and no rehearsal), with 28 participants in each condition.

Participants were tested individually. They were asked to study four routes, one at a time, so that they could later describe the routes to an experimenter. On each trial they were presented with a diagram that showed a complete route on an A4-sized paper (i.e., Figure 1). In order to help them to get familiar with the sequence of steps from the starting point to the destination, participants were told to physically trace the existing route on the map twice with a highlighter. They should trace every step and not to pause at any junctions of the route. Then we removed the diagram. In the pilot test, we asked twenty participants, with five in each condition, to view the existing routes (each for 20 seconds) instead of physically tracing them on the maps twice with a highlighter. However, at least half of the participants in each condition recalled most of the steps in wrong order. One possibility was that they did not process the stimuli as routes that navigated from starting points to destinations. Rather, they might perceive them as lines and arcs that formed various visual patterns. Therefore, we changed our protocol and asked participants to physically trace the existing routes.

Participants then received different instructions for rehearsal in different conditions. In the co-thought gesture condition, participants were told to rehearse the route from the starting point to the destination with their hands. In the drawing condition, participants were instructed to draw the route from the starting point to the destination once on a piece of *blank* A4-sized paper. Participants were told that they were not required to draw the route in the same scale as that shown in the previous diagram. They were also told that neatness of their drawing would not be evaluated. In the hand movement prevented condition, participants were told to visualize or mentally simulate the route sequence from the starting point to the destination once while holding a softball in both hands. We did not control the amount of the time participants spent visualizing the routes as there might be individual variations. We rather controlled the number of the time participants visualized the route by instructing them to visualize it once in each trial. Participants in this condition did not move their arms while holding a softball in both hands. Then they informed the experimenter when they finished visualizing a complete route. In the no rehearsal condition, participants were given an A4-sized paper with randomly selected alphabets printed on random locations of the page. They were told to read the alphabets aloud for 20 seconds in order to prevent them from mentally rehearsing the route. We conducted pilot study and found that on average participants spent 20 seconds on rehearsing a complete route in the hand movement prevented condition. Therefore, we asked participants to read letters aloud for 20 seconds. We also expected that reading letters aloud would not depress participants' performance by interfering their spatial representations because the letters were randomly printed on an A4-sized paper such that they did not form any clear spatial pattern.

Finally, all participants were asked to verbally describe the route from the starting point to the destination from their memory to an experimenter. For example, one would say, “You go up, and then turn right, go down, and move diagonally upwards”.

Before they studied the second route, participants were required to work on a set of mathematics problems for two minutes in order to prevent proactive interference of the directions from the previous route. Then the second diagram was presented and the aforementioned procedures were repeated. The experiment was complete after all four routes were studied. The order of diagrams was randomized across participants. The whole experiment was videotaped. This research was approved by the Institutional Review Board (IRB) at the Chinese University of Hong Kong. Written informed consent were obtained from the participants.

#### Scoring and coding

All videos were displayed by VLC media player. We watched the videos and coded the average amount of time (in seconds) each participant spent rehearsing a complete route (including pauses and self-corrections, if any) across four diagrams in different conditions (except participants in the no rehearsal condition). In the co-thought gesture condition, rehearsal started when participants raised their hands and ended when they had their hands rest on the table or their thighs. In the drawing condition, rehearsal started when participants started drawing on paper and ended when they put down the pen. In the hand movement prevented condition, rehearsal started when participants received the signal from the experimenter and ended when they informed the experimenter that they were done with rehearsal. We also counted the mean number of steps participants rehearsed in the co-thought gesture and drawing conditions.

All spoken responses in the recall phase were transcribed by native English speakers and speech units that contained the descriptions of the steps (e.g., “*go left*”; “*turn right*”) were identified. We then assessed the accuracy of recall by considering how many steps (out of thirteen) were accurately recalled in the speech descriptions in each diagram. A step was considered to be correctly recalled if a participant's verbal description about this step matched its direction *and/or* order in the diagram. A step was considered to be recalled incorrectly if 1) it was omitted; 2) it was mis-ordered which resulted in an inaccurate recall of the direction or 3) it was not actually found in the route. The mean proportion of steps correctly recalled in each diagram was calculated for each participant, which was the number of steps correctly recalled, divided by thirteen (i.e., the total number of steps in the diagram).

We also measured the average amount of time (in seconds) each participant spent on the recall (including all pauses, hesitations, e.g. “*um*”, “*uh*”, “*and you…*”, and self-corrections, e.g., “*you turn left*. *No, sorry, you go up*”) across four diagrams.

Reliability was assessed by having a second coder code a subset (20%) of the data. Regarding the rehearsal and recall duration, we counted the number of trials agreed upon by both coders and divided it by the total number of trials included in the reliability analyses. We considered both coders agreed with each other if the difference in their rehearsal or recall duration was equal or less than 1 second in each trial. Note that we might not accurately code the durations from the videos because of the activities (e.g., rehearsal) may not be visible to an outside observer. For example, participants might be mentally rehearsing even before they started moving their hands. Besides, there was a one-second interval in defining agreement in timing. Therefore, there was a limitation in our measures of rehearsal duration. Inter-rater agreement for the rehearsal duration was 90% (Cohen's Kappa  = .85, *p*<.001) and 85% for the recall duration (Cohen's Kappa  = .83, *p*<.001). We also looked at how many trials in which the number of steps rehearsed in the co-thought gesture and drawing conditions agreed upon by both coders. We found that the inter-rater agreement was 89% (Cohen's Kappa  = .84, *p*<.001). Finally, we examined how many trials in which both coders identified the same number of co-speech gestures produced during verbal recall. The inter-rater agreement was 87% for identifying gestures produced in the recall (Cohen's Kappa  = .85, *p*<.001).

Within each trial, we counted the number of times in which both coders agreed with the accuracy of steps recalled and found that the inter-rater agreement was 90% (Cohen's Kappa  = .88, *p*<.001). Finally, we examined whether both coders coded the same gestures that were co-occurring with particular segments of speech. The inter-rater agreement was 87% for identifying gestures produced in the recall (Cohen's Kappa  = .85, *p*<.001).

### Results

We aimed to examine whether producing co-thought gestures facilitated encoding of spatial information and whether the impact of co-thought gestures was beyond that of drawing. In the paragraphs below, we first reported the amount of time participants spent on rehearsal and the number of steps they rehearsed, followed by their recall performance.

All participants in the co-thought gesture condition gestured when they were rehearsing the routes. Majority of participants used their index fingers to trace the steps in the air (e.g., an index finger extended and moved to the right and then upwards). A few participants use palms instead (e.g., all fingers extended and swiped horizontally and moved downwards). All participants gestured with their right hands. On average, participants in the co-thought gesture condition spent 18.12 seconds (*SD* = 2.32) to rehearse a route. Participants in the drawing and hand movement prevented conditions spent 25.08 seconds (*SD* = 2.78) and 23.14 seconds (*SD* = 1.19) respectively. One-way ANOVA showed that there was a significant difference in the rehearsal duration among different conditions, *F* (2, 81)  = 16.81, *p*<.001. Tukey posthoc tests showed that the time spent rehearsing a complete route in the co-thought gesture condition was less than that in the drawing condition, *p*<.001, but not different from that in the hand movement prevented condition, *p = *ns. The mean number of steps participants rehearsed in the co-thought gesture condition was 11.58 (*SD* = 4.79), and that in the drawing condition was 12.37 (*SD* = 5.43), *t* (54)  = 1.59, *p* = ns. The difference did not reach statistical significance. The number of steps rehearsed was equal to the number of hand movements (in the forms of gestures or drawing) produced during rehearsal.

**Figure 2:**
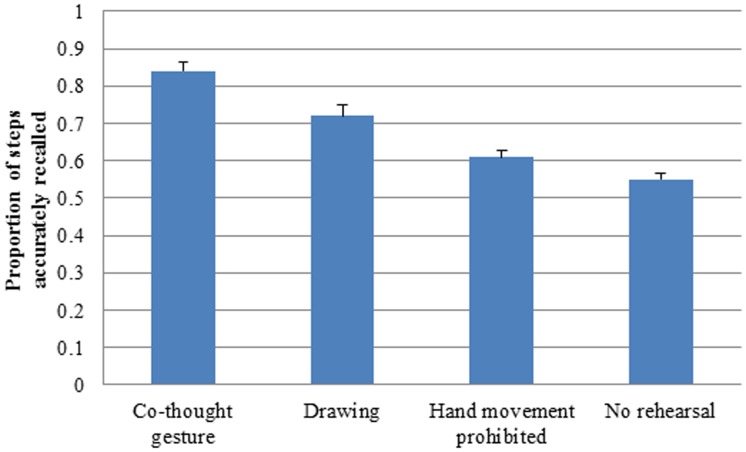
The mean proportion of steps correctly recalled across four trials in the co-thought gesture, drawing, hand movement prevented and no rehearsal conditions.

Our main interest was in whether participants' spatial recall performance differed across conditions. We first examined the proportion of steps accurately recalled. Figure 2 shows the mean proportion of steps correctly recalled in the four conditions. We conducted ANOVA with condition (co-thought gesture, drawing, hand movement prevented, no rehearsal) as the between-subject independent variable and the mean proportion of steps correctly recalled across four trials as the dependent variable. There was a significant effect of condition, *F* (3, 107)  = 5.54, *p*<.001, *η^2^* = .18. Tukey posthoc tests showed the proportion of steps correctly recalled in the co-thought gesture condition was higher than that in the drawing condition, *p*<.01, that in the hand movement prevented alone condition, *p*<.001, and that in the no rehearsal condition, *p*<.001. The proportion of steps correctly recalled in the drawing condition was also higher than that in the hand movement prevented condition, *p*<.01, and that in the no rehearsal condition, *p*<.001. There was no difference between the hand movement prevented and the no rehearsal conditions, *p* = ns.

We found that they recalled comparable number of steps in all the four trials. We conducted repeated measures ANOVA with the condition (co-thought gesture, drawing, hand movement prevented, no rehearsal) as the between-subject independent variable, trial (1^st^, 2^nd^, 3^rd^, 4^th^) as the within-subject independent variable, and the proportion of steps accurately recalled as the dependent variable. There was a significant effect of the condition, *F* (3, 107)  = 6.97, *p*<.001, *η^2^* = .16, no effect of trial, *F* (3, 321)  = .57, *p* = ns, and no interaction, *F* (9, 321)  = .57, *p* = ns. Thus, the recall performance was comparable across the four trials.

Participants in the present study were not given any time limit for recalling the routes. Thus, it was possible that participants in the co-thought gesture condition performed better in the spatial recall because they took more time to recall. Nonetheless, participants spent a comparable amount of time (in seconds) for recalling the routes in all conditions. On average, participants spent 27.82 seconds (*SD* = 3.74) to recall a route in the co-thought gesture condition; 26.34 seconds (*SD* = 2.82) in the drawing condition; 27.58 seconds (*SD* = 3.14) in the hand movement prevented condition; and 29.23 seconds (*SD* = 3.89) in the no rehearsal condition, *F* (3, 107)  = .84, *p* = ns. Therefore, the more accurate recall in the co-thought gesture condition was not attributable to a longer recall time.

Yet, one might contend that participants who traced the step sequence in the air might produce more *co-speech gestures* in the recall phase than those in the other conditions. If so, producing co-thought gestures in rehearsal might have nothing to do with consolidating spatial information, but it might instead induce co-speech gestures when talking, which in turn, enhanced spatial recall. In order to explore this possibility, we calculated the numbers of co-speech gestures per description of step in each condition. We only included co-speech gestures that indicated the directions, e.g., index finger moving up as referring to an upward movement. Gestures that did not convey directional meaning, e.g., speech beats, were excluded from this analysis. Participants produced comparable numbers of co-speech gestures per step across the four conditions, *F* (3, 107)  = 1.82, *p* = ns. They produced.96 (*SD* = .27) co-speech gesture per step in the co-thought gesture condition;.87 (*SD* = .43) in the drawing condition;.95 (*SD* = .38) in the hand movement prevented condition; and.92 (*SD* = .29) in the no rehearsal condition. Thus, our manipulation of experimental conditions did not influence participants' co-speech gesture rates during recall. In addition, we examined correlations between the number of co-speech gestures produced during recall and the proportion of steps accurately recalled in each condition. No significant correlations were found: *r* (26)  = .18, *p* = ns, in the co-thought gesture condition; *r* (26)  = .21, *p*  =  ns, in the drawing condition; *r* (26)  = .19, *p* = ns, in the hand movement prevented condition; and *r* (26)  = .24, *p* = ns, in the no rehearsal condition. Thus, the difference in recall among the conditions was not attributed to the frequency of co-speech gestures during recall.

### Discussion

In Experiment 1, participants who were instructed to gesture or draw the routes on paper during rehearsal recalled more steps than those who were told to visualize the routes and those who did not rehearse the routes. Hence, hand movements produced during rehearsal, either in the forms of gestures or drawing, facilitated encoding of spatial information, which in turn enhanced subsequent recall. Interestingly, participants in the co-thought gesture condition had better recall than those in the drawing condition. As a result, co-thought gestures seem to be more powerful than drawing in encoding spatial information for recall.

However, two issues remain unaddressed in Experiment 1. First, it is not clear *why* participants in the co-thought gesture condition recalled more steps than those in the drawing condition. There are at least three possibilities. One possibility is that the rehearsal time in the co-thought gesture condition was significantly shorter than that in the drawing condition. Interestingly, the number of steps rehearsed in the co-thought gesture condition was comparable to that in the drawing condition. In other words, longer rehearsal time did not lead to an increase in the number of steps rehearsed. Rather, an overly long rehearsal time might cause one to lose attention on the steps to be remembered, which in turn increases the chance of forgetting and worsens the recall performance [27]. However, there was no significant difference in the rehearsal time between the co-thought gesture condition (and the drawing condition) and the hand movement prevented condition. But the recall performance in the co-thought gesture condition (and the drawing condition) was better than that in the hand movement prevented condition. As a result, shorter rehearsal time in the co-thought gesture condition could not explain its better recall performance as compared to other conditions.

Another possibility is that participants could gesture freely in a three-dimensional space in the co-thought gesture condition whereas those in the drawing condition drew in a two-dimensional space (i.e., on paper). Gesturing in a three-dimensional space might impose less constraints on hand movements than drawing in a two-dimensional space, and thereby maximizing the beneficial effect of rehearsal and enhancing the recall.

Alternatively, producing co-thought gestures in the air consolidated spatial sequence in mental representation better than drawing on paper. Gesturing spatial sequences in the air leaves no visible trail whereas drawing does. Therefore, participants who rehearsed with gestures might have to maintain an active image of a sequence in their mental representation, thereby constructing a rich internal representation of the spatial sequence. In contrast, when participants were drawing a sequence on paper, they did not have to hold the sequence actively in their mind (as they were able to see it clearly on the paper).

In order to investigate which factor (rehearsing freely in a three-dimensional space vs. better consolidation of spatial information) could explain the better recall performance in the co-thought gesture condition, we asked a group of participants in Experiment 2 to gesture on paper (co-thought gesture on paper condition). Like drawing, gesturing on paper involves hand movements in a two-dimensional space. If the better recall performance in the co-thought gesture condition was attributed to gesturing in three-dimensional space, then the facilitation effect of gesture compared to drawing would disappear when participants were asked to gesture on paper. Participants in the co-thought gesture on paper condition should recall comparable number of steps to those in the drawing condition. However, if the better recall performance in the co-thought gesture condition was due to the fact that gesturing does not leave any visible trail, then the facilitation effect of gesture would still remain significant. Participants in both co-thought gesture conditions (in the air and on paper) should recall more steps than those in the drawing condition.

Another issue remained unaddressed in Experiment 1. We evaluated participants' spatial learning outcome by looking at the accuracy rate in their verbal recall. Therefore, one might contend that the better recall performance in the co-thought gesture condition might be due to the recall modality rather than co-thought gestures per se. This could be manifested in three ways. First, the production of co-thought gestures during rehearsal might activate the language processes that were later involved in the route description. Second, drawing on paper was a perceptual activity as well as a motor activity, and thus it was likely to be incongruent with verbalization. According to the work done by Schooler and colleagues [28]–[30], verbally describing a nonverbal stimulus can impair subsequent attempts at identification of the stimulus. Finally, the rehearsal procedures in the drawing and hand movement prevented conditions were much less similar to the recall phase of verbally describing the routes than that in the co-thought gesture condition. Specifically, in the drawing and hand movement prevented conditions, participants were no longer holding a pen and paper, or a ball, during recall. In contrast, those in the co-thought gesture condition were doing the same thing throughout the phases of the study. Any similarity would favor the participants in the co-thought gesture condition. In order to exclude the possibility that our findings were affected by the verbal nature of our outcome measure, we asked participants in Experiment 2 to engage in a nonverbal task, which was, reconstructing the routes with sticks.

## Experiment 2

### Method

#### Participants

One hundred and forty English-speaking undergraduates (65 men, age range: 19–21 years) participated in this experiment in order to fulfill course requirements. All of them had correct or correct-to-normal vision. One hundred and thirty-nine participants were right-handed.

#### Stimuli

The stimuli in Experiment 2 were the same as those in Experiment 1.

#### Procedures

Participants were randomly assigned to one of the five conditions (co-thought gesture in the air, co-thought gesture on paper, drawing, hand movement prevented, no rehearsal), with 28 participants in each condition.

The procedures in the co-thought gesture in the air, drawing, hand movement prevented, and no rehearsal conditions were the same as those in Experiment 1. Regarding the co-thought gesture on paper condition, participants were told to rehearse the route from the starting point to the destination on paper with their hands.

Finally, all participants were given thirteen sticks (each three inches long) and told to reconstruct the route sequence on a table all the way from the starting point to the destination. They were told that they were not required to reconstruct the route in the same scale as that shown in the diagrams.

As in Experiment 1, participants worked on a set of mathematics problems for two minutes before they studied the second route. The experiment was completed after all four routes were studied. The order of diagrams was randomized across participants. The whole experiment was videotaped.

#### Scoring and coding

As in Experiment 1, we watched the videos and coded the average amount of time (in seconds) participants spent rehearsing a complete route (including pauses and self-corrections, if any) across four diagrams in different conditions (except the no rehearsal condition). We also examined the mean number of steps participants gestured (in the air or on paper) or drew on paper in order to ensure that participants rehearsed comparable number of steps in these three conditions.

We then assessed the accuracy of recall by counting from videos how many steps (out of thirteen) were accurately reconstructed by sticks for each diagram. We used the same assessment criteria as in Experiment 1 and calculated the mean proportion of steps correctly reconstructed in each diagram for each participant. We also measured the average amount of time (in seconds) each participant spent reconstructing each route (including all pauses and hesitations).

Reliability was assessed by having a second coder code a subset (20%) of the data. Inter-rater agreement was 98% (Cohen's Kappa  = .95, *p*<.001) for measuring the time spent on rehearsal; 91% (Cohen's Kappa  = .88, *p*<.001) for identifying the number of steps rehearsed in the co-thought gesture in the air, co-thought gesture on paper, and drawing conditions; 95% for determining the accuracy of steps reconstructed (Cohen's Kappa  = .92, *p*<.001); and 100% for determining the duration of reconstruction (Cohen's Kappa  = 1, *p*<.001).

### Results

In Experiment 2, we aimed to examine 1) whether gesturing on paper would be as effective as gesturing in the air but more effective than drawing on paper; and 2) whether the beneficial effect of gestures was even found in a nonverbal recall task.

As in Experiment 1, participants in the co-thought gesture in the air condition gestured when they were rehearsing the routes and most of them used their index fingers. All but one participant gestured with their right hands. On average, participants in the co-thought gesture in the air condition spent 17.86 seconds (*SD* = 2.32) to rehearse a route. Participants in the co-thought gesture on paper condition also gestured with their index fingers tracing the routes on paper during rehearsal. All of them gestured with their right hands. They spent 16.52 seconds (SD = 2.78) to rehearse a route. Participants in the drawing and hand movement prevented conditions spent 24.81 seconds (*SD* = 3.14) and 19.18 seconds (*SD* = 1.19) respectively. One-way ANOVA showed that there was a significant difference in the rehearsal duration among different conditions, *F* (3, 108)  = 14.24, *p*<.001. Tukey posthoc tests showed that the time spent rehearsing a complete route in the drawing condition was significantly longer than that in the co-thought gesture in the air, *p*<.001, and that in the co-thought gesture on paper condition, *p*<.001, and hand movements prevented condition, *p*<.002. There was no difference between the two co-thought gesture conditions, and between them and hand movement prevented conditions, *ps* = ns.

The mean number of steps participants rehearsed in the co-thought gesture in the air condition was 11.83 (*SD* = 3.54), and that in the co-thought gesture on paper condition was 12.06 (*SD* = 3.17), and that in the drawing condition was 11.91 (*SD* = 5.43), *t* (81)  = .97, *p* = ns. Thus, participants in all three conditions rehearsed comparable numbers of steps. As in Experiment 1, the number of steps rehearsed was equal to the number of hand movements (in the forms of gestures or drawing) produced during rehearsal.

**Figure 3:**
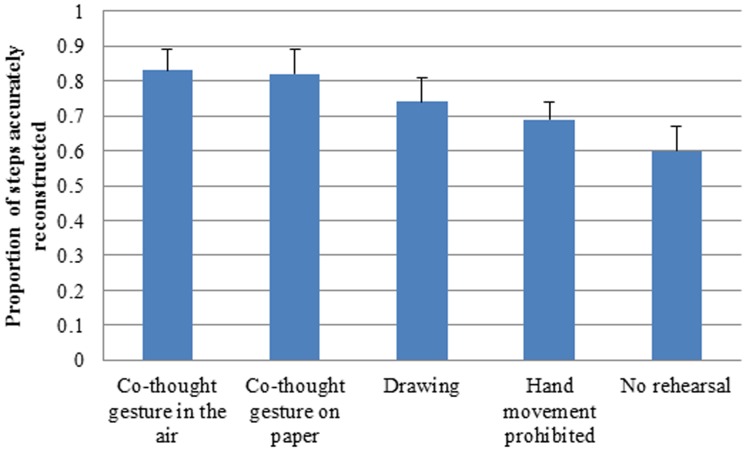
The mean proportion of steps correctly recalled in the co-thought gesture in the air, co-thought gesture on paper, drawing, hand movement prevented and no rehearsal conditions.

We then examined the proportion of steps accurately reconstructed, which was our main interest. Figure 3 shows the mean proportion of steps correctly reconstructed in the five conditions. We conducted ANOVA with condition (co-thought gesture in the air, co-thought gesture on paper, drawing, hand movement prevented, no rehearsal) as the between-subject independent variable and the proportion of steps correctly reconstructed as the dependent variable.

There was a significant effect of condition, *F* (4, 135)  = 46.81, *p*<.001, *η^2^* = .19. Tukey posthoc tests showed the proportion of steps correctly reconstructed in the co-thought gesture in the air condition was higher than that in the drawing condition, *p*<.001, that in the hand movement prevented condition, *p*<.001, and that in the no rehearsal condition, *p*<.001. These results replicated those in Experiment 1. The proportion of steps correctly reconstructed in the co-thought gesture on paper condition was higher than that in the drawing condition, *p*<.001, that in the hand movement prevented condition, *p*<.001, and that in the no rehearsal condition, *p*<.001. There was no significant difference between the two co-thought gesture conditions, *p* = ns.

As in Experiment 1, the proportion of steps correctly reconstructed in the drawing condition was higher than that in the hand movement prevented condition, *p*<.02, and that in the no rehearsal condition, *p*<.001. However, now participants in the hand movement prevented condition reconstructed more steps than those in the no rehearsal condition, *p*<.01.

On average, participants spent a comparable amount of time (in seconds) in reconstructing a route in all conditions: 28.32 seconds (*SD* = 3.51) in the co-thought gesture in the air condition; 27.54 seconds (SD = 2.98) in the co-thought gesture on paper condition; 29.48 seconds (*SD* = 3.19) in the drawing condition; 28.38 seconds (*SD* = 3.29) in the hand movement prevented condition; and 30.26 seconds (*SD* = 3.41) in the no rehearsal condition, *F* (4, 135)  = .78, *p* = ns. Therefore, the greater reconstruction accuracy in both co-thought gesture conditions was not attributed to the time spent on recall.

### Discussion

In the second experiment, we found that gesturing on paper facilitated encoding of spatial information and this facilitation effect was the same as that in the gesturing in the air condition but stronger than that in the drawing condition. These findings have two implications. First, the facilitation effect of co-thought gestures could not be explained by gesturing in a three-dimensional space. Rather, co-thought gestures might more efficiently consolidate spatial representation than drawing. Second, producing co-thought gestures was found to be effective for verbal as well as nonverbal spatial recall. Therefore, the facilitation effect found in Experiment 1 was not attributable to the verbal nature of our outcome variable.

## General Discussion

### Co-thought gestures facilitate encoding of spatial information

The findings in the present study contribute to the field of gesture research in a way that co-thought gesture, as a kind of representational action, is an effective technique in facilitating encoding of spatial information. There are very few studies to date that show the role of gesture in encoding spatial information or spatial learning in general, despite the fact that gesture itself is spatial in nature [8] and it often represents visuo-spatial information (e.g., [8], [12], [31]–[33]). Of a few studies, Chu and Kita [20] found beneficial roles of co-thought gestures in mental rotation task; Ehrlich, Levine, and Goldin-Meadow [17] reported that frequency of co-speech gestures is positively associated with children's performance in the mental rotation task.

Participants in the co-thought gesture condition as well as those in the hand movement prevented condition were encoding spatial sequences mentally. This was because they could not see the trails they were rehearsing; they would have to maintain the steps actively in their memory. However, unlike participants in the hand movement prevented condition who did not move their hands and arms, those in the co-thought gesture conditions gestured. Their co-thought gestures might offload the mental representation of routes, which in turn reduces the chance of forgetting [20], [34]. These gestures might also help participants to create their own mental images of the routes, which are known to lead to enhanced memory [35].

However, one might contend that participants in the co-thought gesture (and drawing) condition performed better than did those in the hand movement prevented condition because of the interference with the rehearsal in the hand movement prevented condition rather than the cognitive enhancement from gestures. This is because additional experimental instructions for asking them not to gesture might impose an additional working memory load to a degree that it might interfere with spatial processes [36]. Our study cannot exclude this possibility. However, there is no empirical evidence showing that similar sort of experimental instructions would impair performance in spatial tasks. Chu and Kita [20] (Experiment 2) did not find any difference in the performance between the gesture-allowed group and the gesture-prohibited group in the mental rotation task. Therefore, instructing participants not to gesture might not interfere with participants' spatial thinking.

### Co-thought gestures exert more influence on spatial information encoding than drawing

It is of particular interest, our findings showed that producing co-thought gestures brought a greater impact on encoding and retaining spatial information than drawing on paper. Participants who gestured the routes (in the air or on paper) recalled more steps than did those who drew them on paper. Then why was the recall performance in the co-thought gesture condition better than drawing condition? Indeed, drawing routes on paper in the drawing condition might also help participants to offload their spatial representation of steps. However, when they drew the sequences on paper, they did not have to represent these sequences mentally as they could see the steps.

Our results are also in line with Novack et al.'s [24] where they reported that learning how to solve mathematical-equivalence problems with gesture was more effective in promoting generalization than learning the same concept with direct action on objects. They proposed that gesturing helps learners to focus attention on the relevant aspects of concepts and away from irrelevant aspects, hence facilitating processing and generalization of knowledge. Based on this view, participants in the co-thought gesture condition might only produce essential hand movements that are directly relevant to remembering the steps. In contrast, participants in the drawing condition might put additional but irrelevant effort in drawing the routes, although they were told that neatness was not required.

Taken together, gesturing routes might be a more effective rehearsal method than drawing. Specifically, producing co-thought gestures might help participants to offload immediate representation while building a richer mental representation of the path [19], [37]–[40]. The findings of this study advance our understanding about the effects of different kinds of hand movements, specifically hand movements with and without leaving physical visible traces, on spatial learning. While hand movements in general gave better recall than the movement-prevented condition, gesturing was significantly more helpful than drawing.

However, one might argue that producing co-thought gestures promoted spatial learning because it was more novel and more effortful than merely drawing the routes on paper. Yet, participants in the co-thought gesture conditions spent less time on rehearsing the routes than did those in the drawing condition in both experiments, implying that they did not pay extra efforts during rehearsal (note that participants in the co-thought gesture and drawing conditions rehearsed similar number of steps). In addition, the amount of time spent on rehearsal in the co-thought gesture conditions was comparable to that in the hand movement prevented condition. Hence, gestures might require relatively little effort to produce (see also [22], [34]).

On the other hand, it is also possible that participants in the co-thought gesture conditions were more likely to speak to themselves (i.e., subvocalizing), given gestures are always accompanying speech in a natural discourse, than those in the drawing condition. If so, participants in the co-thought gesture conditions might encode spatial information in two modalities – gesture and subvocalized speech, which would in turn strengthen their spatial memories. However, we did not find the beneficial effects of encoding spatial information in both spoken and gestural modalities on learning and memory in our recent study. Currently we are conducting a study that investigates whether producing gestures while thinking silently (co-thought gestures) and speaking facilitation effect was stronger than that of spatial language, which is the most commonly used symbol system in representing spatial information. We found that participants who were encouraged to move their hands during rehearsal had the best recall. Of more importance, this result was found regardless of whether participants rehearsed with spatial language. These findings further corroborate the beneficial nature of gesture in processing spatial information.

### The facilitation effect of co-thought gestures holds for verbal and nonverbal recall modalities

Intriguingly, the facilitation effect of co-thought gestures is robust in both verbal and nonverbal recall. This finding has two implications. First, it indicates the fact that participants in the co-thought gesture condition outperformed those in other conditions (in Experiment 1) is due to the facilitation effect of gestures rather than interference possibly found in the other conditions. One kind of interference was possibly caused by the differences between the rehearsal and recall procedures in the drawing and hand movement prevented conditions. Another kind of interference might be caused by the verbal overshadowing effect where asking participants to verbally describe the routes after drawing in the drawing condition might impair their recall performance [28]. However, in Experiment 2, we asked participants to reconstruct the routes with sticks. Therefore, the rehearsal procedures in the co-thought gesture conditions (also the other conditions) were different from the recall procedures. The verbal overshadowing effect in the drawing condition was also avoided. Our findings in Experiment 2 were consistent to those in Experiment 1: participants who produced co-thought gestures outperformed those in other conditions. As a result, it suggests that the better recall performance in the co-thought gesture condition was not attributed to the interference possibly found in other conditions but the facilitation effect of co-thought gestures.

Second, the recall performance is independent of modality. As discussed earlier, producing co-thought gestures might activate the language processes involved in route descriptions. This is possibly because speech and gestures commonly co-occur and are thought to have overlapping (or interacting) mental representations [8], [9]. Thus, one might contend that the benefit of gesture over drawing in Experiment 1 could be due to the verbal recall. However, we still found the facilitation effect of co-thought gestures in Experiment 2 where participants were asked to reconstruct the routes with sticks. As a result, asking participants to verbally describe the routes in Experiment 1 did not actually favor those in the co-thought gesture condition.

Taken together, the findings in both Experiment 1 and Experiment 2 provide converging evidence that encoding spatial information using co-thought gestures strengthens spatial recall in both verbal and nonverbal modalities.

### Future studies

The present study focused on the role of co-thought gestures in improving encoding of spatial information. In future, we should also investigate whether producing *co-speech gestures* while verbally rehearsing the routes would enhance encoding and retention as well, and compare its effect with that of co-thought gestures. Chu and Kita [18] suggested that these two types of gestures originate from a common system that is independent of speech production process and that their behaviors show close parallelism. Accordingly, producing co-thought and co-speech gestures should yield the same level of facilitating effect on learning spatial sequence.

### Conclusions

To conclude, producing co-thought gestures is an effective method for encoding spatial information. In addition, co-thought gesture is more powerful than drawing in facilitating spatial information encoding. Producing co-thought gestures allows us to construct the spatial information and retain it in our memory with relatively little effort. Further research could address whether such encoding method can be applied to other spatial tasks and how long its mnemonic effect is. Based on the findings in this study, however, we could start practicing moving our fingers in the air when we are learning a direction in a new environment.
